# Peripheral Vascular Abnormalities in Anorexia Nervosa: A Psycho-Neuro-Immune-Metabolic Connection

**DOI:** 10.3390/ijms22095043

**Published:** 2021-05-10

**Authors:** Maria Maddalena Sirufo, Lia Ginaldi, Massimo De Martinis

**Affiliations:** 1 Department of Life, Health and Environmental Sciences, University of L’Aquila, 67100 L’Aquila, Italy; maddalena.sirufo@gmail.com (M.M.S.); lia.ginaldi@cc.univaq.it (L.G.); 2 Allergy and Clinical Immunology Unit, Center for the Diagnosis and Treatment of Osteoporosis, AUSL 04 Teramo, 64100 Teramo, Italy

**Keywords:** anorexia nervosa, neuroendocrine dysregulation, cytokines, cardiovascular diseases, capillaroscopy, nailfoldcapillaroscopy, microvascular damage, microcirculation, eating disorders, autoimmunity

## Abstract

Immune, neuroendocrine, and autonomic nervous system dysregulation in anorexia nervosa lead to cardiovascular complications that can potentially result in increased morbidity and mortality. It is suggested that a complex non-invasive assessment of cardiovascular autonomic regulation—cardiac vagal control, sympathetic vascular activity, and cardiovascular reflex control—could represent a promising tool for early diagnosis, personalized therapy, and monitoring of therapeutic interventions in anorexia nervosa particularly at a vulnerable adolescent age. In this view, we recommend to consider in the diagnostic route, at least in the subset of patients with peripheral microvascular symptoms, a nailfold video-capillaroscopy as an easy not invasive tool for the early assessing of possible cardiovascular involvement.

## 1. Introduction

Anorexia nervosa (AN) is a severe psychiatric disorder, which mainly affects females, associated with frequent medical complications and characterized by malnutrition and starvation [1]. Genetic and environmental factors influence the disease. The study by Duncan et al. concluded that AN is a complex heritable phenotype with large and significant genetic correlations with both psychiatric phenotypes and metabolic traits and identified one genome-wide significant locus on chromosome 12 in a region harboring a previously reported type 1 diabetes and autoimmune disorder locus [2]. A reconceptualization of AN as both a psychiatric and metabolic disorder has been encouraged by significant genetic correlations with several metabolic phenotypes and psychiatric personalities such as lipid metabolism, glucose metabolism, neuroticism, and schizofrenia. Furthermore, epigenetics might help untagle the molecular genetic pathways participating in the development and progression of the disease. To elucidate the role of disease-associated epigenetic dysregulation in specific genetic and environmental contexts, integrated analyses are emerging that incorporate detailed environmental data with genotypic, epigenetic, and transcriptomic data [3,4,5]. Adults with AN show a dysregulation of the hypothalamic-pituitary-adrenal-axis activity (HPA-axis) and childhood trauma exposure contribute to this dysregulation [6].

Conditions attributable to purging, malnutrition, and weight loss are the frequent causes of medical complications. Almost all organs and systems are affected in the course of the disease, and sometimes some abnormalities that it causes persist even when it recovers. Among others, the occurrence of cardiovascular complications characterize anorexia nervosa. The cardiovascular system is heavily involved as bradicardia, hypotension, arrhytmias, prolongation of QT interval, and peripheral vascular abnormalities may occur. Morbidity and mortality in AN are mainly due to cardiovascular complications and the pathophysiological mechanism leading to higher cardiovascular risk is still unclear. Dysregulation between peripheral vasoconstriction and vasodilatation, clinically appearing with Raynaud’s phenomenon (RP), is reported in AN [7].

## 2. Pathogenesis of RP

Peripheral and central neurogenic mechanisms and endothelium derived mediators regulate the vascular tone of cutaneous vessels thus playing a leading role in thermoregulation. Sympathetic noradrenergic and sympathetic cholinergic nerves mediate vasoconstriction and vasodilation of blood vessels in human skin. There is evidence for a role of the central nervous system in the underlying defect of RP together with a localized failure. The exaggerated vasoconstriction of RP is at least partially explained with the increased number of adrenergic receptors on the vascular smooth muscle cells, further compromised in the presence of associated vascular injury or obstruction. Thus, the vasoconstrictive responses to centrally mediated release of norepinephrine is further enhanced. Blood flow to the deep tissue and skin of the fingers and normal thermoregulation can be easily disrupted by the fragile balance of interacting pathways.

RP is an acrosyndrome (an altogether assemblage of symptoms and signs in the acral body areas, such as nose, fingers, toes, ears, etc.), with a complex of symptoms, derived from an alteration of vasoconstriction/vasodilation mechanisms: its diagnostic work-up should include a nailfold capillaroscopy [8,9] and antinuclear antibody testing [10]. RP may or may not be associated to autoimmune disease and a progression of nailfold capillaroscopic pattern is a predictor of evolution [11,12,13].

## 3. RP, Metabolism, and Oxidative Status

In adolescents and children with RP an interestingly relationship between lipid metabolism, the concentration of biomarkers such as high-sensitivity c-reactive protein, endothelin-1, sE-selectin, and the qualitative abnormalities found on nailfold capillaroscopy is reported [14]. Metabolic disorders may influence vascular reactivity without direct injury to thermoregulatory vessels. AN among several other diseases may be associated with RP probably due to disrupted neurogenic regulation and vascular injury [13]. Protein undernourished patients show a higher level of free radicals and lower antioxidants compared to adequately nourished people. Mitochondrial dysfunction and oxidative stress in peripheral blood leukocytes at the level of the mitochondrial complex I are observed in anorexia nervosa also due to ROS and proinflammatory cytokines released by immune cells. Some of these cytokines mediate weight loss through catabolizing the energy reserve and reducing food intake. Oxidative stress and endothelial and mitochondrial dysfunction may result from the joint action of ROS and proinflammatory cytokines. Anorexia increases ROS production with activation of inflammation pathways, which increase in the endothelia the production of adhesion molecules and consequently activate immune cell attachment, migration, and infiltration to inner organs [15]. Chronically increased levels of oxidative stress are observed in both primary and secondary RP patients.

## 4. AN, Inflammation, and Immunity

The immune system and central nervous system have a vital means of communication through cytokines produced by immune cells. Among these, tumor necrosis factor alpha (TNF-α) possesses manifold metabolic and immunological activities: inhibiting lipoprotein lipase and suppressing food intake. One of the leading types of inflammatory cells are represented by polymorphonuclear leucocytes (PMN) that after activation release proinflammatory molecule and reactive oxygen species (ROS) contributing to endothelial dysfunction. The interaction of adhesion molecules expressed on endothelial cells and leucocytes is crucial for the accumulation of these latter in the vessel wall, one of the fundamental events underlying cardiovascular diseases. The atherosclerotic process sees several cellular adhesion molecule and pro-inflammatory cytokines involved such as E-selectin, vascular cell adhesion molecule 1, intercellular adhesion molecule 1, interleukin 6, and TNF-a. There is a correlation between AN and a proinflammatory state, leucocyte–endothelium interactions, cytokines and adhesion molecules involved. Eating disorders may show endothelial dysfunction due to hyperglycemia or eating restriction resulting in leucocyte recruiting. The study by Víctor VM et al. showed an increased level of the vascular cell adhesion molecule 1, interleukin 6, and TNF-α in anorexic subjects. They demonstrated endothelium–leucocytes interactions due to the inflammatory state of anorexia that may be at least in part responsible of the increased vascular dysfunction observed in these patients [16].

## 5. RP and Nutritional Status

An increased susceptibility to RP could be hypothesized in low body weight people. Recently, Abdulle et al. [17] investigated the association of RP with low body weight, involuntary weight loss, and dietary restrictions. They showed that a low-fat diet is associated with RP in women while in both women and men, an increased risk of RP is significantly associated with prior involuntary weight loss and low body weight [17]. Literature data have highlighted a correlation and association (more than causation) between nutritional status and RP. In fact, most authors have basically recognized a dysfunction of the psycho-neuro-endocrine-immune (PNEI) network at the root of RP and AN [18].

**Figure 1:**
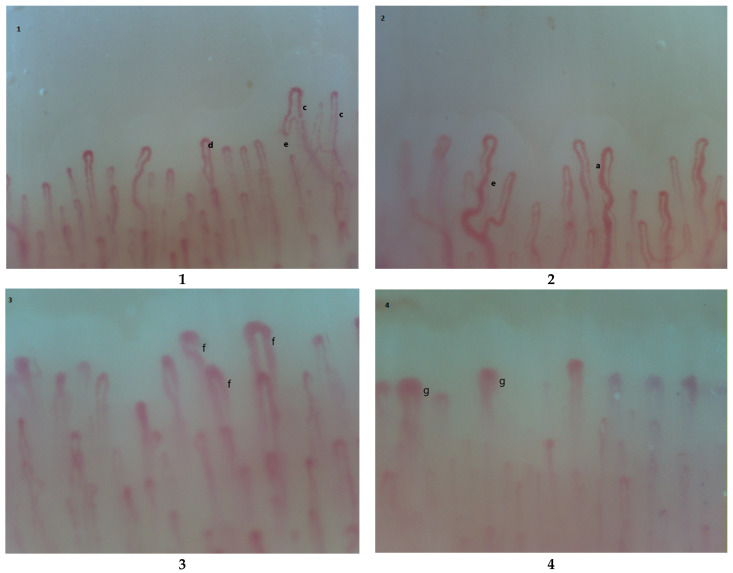
Possible capillaroscopic findings in AN [8,9]. **1**. Aspecific pattern: c. plasma gaps, d. apical ectasias(dilatation), e. morphological anomalies; **2**. Aspecific pattern: a. increase in the afferent/efferent capillar loop ratio, e. morphological anomalies (crossings and tortuosity); **3**. Early scleroderma pattern f. capillary ectasias; **4**. Early scleroderma pattern g. megacapillaries.

It was hypothesized that a person’s susceptibility to RP could be increased by substantial weight loss. The latter could lead to a loss of subcutaneous and perivascular adipose tissue with an increase of vasospastic events. In fact the balance between endothelium dependent vasoconstrictor and vasodilator substances, such as endothelin-1 and nitric oxide, are altered by perivascular adipose tissue [19]. Regarding the relationship between AN and RP, and its role on microvascular damage, a capillaroscopic study of anorexic subjects affected by RP revealed an early scleroderma pattern in 59% of patients. This capillaroscopic picture is also described in 55% of patients with RP and a past history of AN. This study also pointed out that only 6% of patients with primary RP and none with AN alone compared to AN+RP had an early scleroderma pattern. These findings outline an association between early scleroderma pattern and RP in anorexic patients [20]. In other words, in young AN patients, or individuals with AN history and concomitant RP, capillaroscopic abnormalities falling within the spectrum of early scleroderma pattern might be detected (Figure 1). Notably, previous studies have found an association between microvascular abnormalities assessed by nailfold capillaroscopy and macrovascular peripheral endothelial dysfunction evaluated using brachial artery flow-mediated dilatation [21,22]. Moreover, Palova et al. reported that flow mediated vasodilatation is decreased in AN patients compared to the healthy subjects [23]. However, the association between RP and microvascular peripheral endothelial dysfunction is not established yet. Although the peripheral microvascular dysfunction in patients with RP is examined using NC in clinical practice, this method does not reflect nitric oxide-related function [21,24]. Thus, microvascular peripheral endothelial dysfunction in these patients can be also measured peripherally and non-invasively by assessing reactive hyperaemia index using peripheral arterial tonometry [21,25]. The microvascular damage assessed by nailfold capillaroscopy in combination with additional assessment of non-invasive macro- and microvascular peripheral (dys)functions may detect the earliest peripheral vascular abnormalities in anorexia nervosa, and thus contribute to early diagnosis, personalized prevention, and therapy.

## 6. AN and Autonomic Dysfunction

Several autonomic nervous system dysregulation are potentially involved in cardiovascular diseases of anorexia nervosa and, among these, a lack of dynamic adaptability characterized by autonomic imbalance. Peripheral vascular abnormalities caused by a dysregulation between peripheral vasoconstriction and vasodilatation have been described among cardiovascular complications of anorexia nervosa [20]. In this context patients affected by anorexia nervosa may suffer from Raynaud Phenomenon and exhibit nailfold capillaroscopic findings typical of connective tissue diseases. It can be hypothesized that the microvascular damage can contribute to cardiac involvement and a capillaroscopic study may help preventing cardiovascular involvement [26] also in anorexia nervosa. Sekaninova N. et al. suggest that complex non-invasive assessment of cardiovascular autonomic regulation—cardiac vagal control, sympathetic vascular activity, and cardiovascular reflex control—could represent a promising tool for early diagnosis, personalized therapy, and monitoring of therapeutic interventions in anorexia nervosa particularly at a vulnerable adolescent age [27]. Early diagnosis of AN is a critical prognostic factor and heart rate variability may provide a unique assessment for the presence of AN being markedly and consistently altered in these patients [28,29].

The renin-angiotensin system, endothelin, nitric oxide, and reactive oxygen species, all relevant factors regulating vascular functions, also critically influence the sympathetic nervous system that is known to have a leading role in the regulation of the cardiovascular system. The activity of the sympathetic nervous system and the endothelial function have a reciprocal relationship as shown by indirect evidence [30]. In the etiology and pathophysiology of immune-mediated inflammatory diseases, a neural-immune cross-talk mediate chronic systemic inflammation comorbid with stress pathway dysregulation. A significant contributor in the pathophysiology of immune-mediated inflammatory diseases is the sympathetic dysfunction. Chronic inflammation represents a risk factor for autoimmunity for the possible exposure of self-antigens from tissue damage. Presentation and processation of tissue antigens in an inflammatory environment could break tolerance and induce autoimmunity [31]. In adolescents with AN, an insufficient sympathetic cardiovascular control was found, and girls with AN showed signs of early arteriosclerotic damage [32].

## 7. The Metabolic-Neuro-Immune Cross-Talk

Immune, neuroendocrine, and autonomic nervous system dysregulation in anorexia nervosa lead to cardiovascular complications that can potentially result in increased morbidity and mortality.

Recently, Sekaninova et al. reviewed evidence of abnormalities in the immune system and dysfunction in multiple neuroendocrine axes that togheter may help to explain the link between AN and its cardiovascular complications [27]. Various neurological mechanisms are involved in the pathogenesis of AN to which the intricate relation of its psychological component also contribute. In the feeding behavior of anorexics, the corticotrophin-releasing factor, opioids, ghrelin, and leptin mechanisms that have shown to overlap at the hypothalamo-pituitary-adrenal axis, operate collectively to demonstrate the underlying physiological and psychological changes [33].

Much evidence suggests that there is a brain-metabolism-immunity-inflammation axis, connecting in a circular way anorexia, impaired microcirculation and autoimmune disorders. There are pathophysiological and clinical similarities between these disorders, which connect them to each other. Rantala MJ et al. [34] suggest a psychoneuroimmunological model for eating disorders considering that chronic stress may cause autoimmune diseases [35], neuroinflammation increase during autoimmune and autoinflammatory diseases [36], stress responsivity increase as a consequence of immune activation [37], and gut microbiota dysbiosis may lead to the onset of autoinflammatory diseases [38,39,40]. A growing body of literature supports a shared pathophysiology between eating disorders and immune system dysfunction that may suggest a role of immune-mediated interventions in treatment [41]. The immune system and the nervous system provide regulation and defense mechanisms and experimental evidence reveal that they communicate with each other in coordinated mechanisms also triggered by inflammation and autoimmune derangements [42,43].

The identification of individuals with secondary RP, at risk for developing connective tissue disease as well as cardiovascular disease, can be achieved with an early detection of peripheral endothelial dysfunction, using a nitric oxide-dependent non-invasive method [21]. An altered immune function deregulate neurotransmitters [44], neuropeptides [45], and neurohormones responsible for development and self-maintenance of eating disorders and eating-related maladaptive behaviors [46]. Food intake and appetite may be influenced by pro and anti-inflammatory cytokines and vice versa [47,48]. Several studies on the matter have produced divergent results, leaving us to speculate a more complex role of these molecules. Elevated levels of proinflammatory cytokines such as IL-6 and TNF-α are reported in anorexia nervosa, and a peripheral proinflammatory state may result in chronic neuroinflammation. Altered cytokines production may contribute to the inflammation-linked increased risk of cardiovascular complications in anorexia nervosa patients. A specifically increased production of cytokines, chemokines, and endothelial adhesion molecules was observed in affected cardiac tissues [49]. Evaluation, in individuals with eating disorders and depressive comorbidity and dysfunctional eating behaviors at opposite ends of the BMI range, of the serum levels of cytokines, pro-inflammatory (IL-1-α, IL-1β, IL-2, IL-4, IL-6, IL-8, interferon-γ, TNF-α, monocyte chemoattractant protein 1), anti-inflammatory (interleukin IL-10), and growth factors ( vascular endothelial growth factor and epidermal growth factor) showed that IL-1α, IL-10, EGF, and IFN-γ are altered in individuals with AN and binge eating disorders. Potential contributors to the immune system dysregulation reported in these patients are a stress-related chronically activated HPA axis and sympathetic nervous system, an increased oxidative stress, and changes in the intestinal microbiota. In anorexia nervosa as well as in other psychiatric disorders a peculiar inflammatory profile is described together with the possible presence of autoantibodies against neuropeptides that regulate appetite [50,51].

## 8. Autoimmunity and Eating Disorders

Research suggests autoimmune processes to be involved in psychiatric disorders. The study by A. Raevuori et al. suggests a contribution of immune-mediated mechanisms in the development of eating disorders, in fact in patients suffering from these conditions, an increase of autoimmune diseases is observed [52]. Autoimmune diseases and eating disorders give each other a mutual increased risk [53]. An increased risk for eating disorders is observed in children and adolescent affected by autoimmune and autoinflammatory disorders. The role of a bidirectional immune dysfunction in this context is to be investigated and arouses much interest. The association of autoinflammatory and autoimmune diseases to eating disorders is observed in both girls and boys, but appear stronger in males. Probands in families with autoimmune and autoinflammatory diseases are more likely to develop an eating disorder [40]. However, the certain fact is that there is an association between autoimmune disease, anorexia, and RP, and the root psychiatric disturbance is to be located within PNEI vision.

The presence of clinical (Raynaud’s phenomenon) and instrumental (videocapillaroscopic) peripheral vascular alterations typical of an autoimmune disorder such as systemic sclerosis arouses great interest and raises several questions. As acknowledged, in fact, there is a bidirectional relationship between autoimmunity and anorexia nervosa. Furthermore, the mechanisms of damage to the microcirculation and the altered microvascular regulation are also mediated by the immune system and are supported by a chronic inflammatory process. The intriguing hypothesis that autoantibodies could induce RP has been investigated and yielded controversial results [53,54]. An increased risk of autoimmune diseases has been described in eating disorder patients [52]. In the course of systemic sclerosis, the prototype of autoimmune disease associated with RP, the nutritional flow to the skin is severely compromised by a reduction in the microvasculature together with structural changes in digital arteries. In systemic sclerosis, there is evidence of direct immune-mediated injury causing endothelial cells dysfunction. Involved vessels show an endothelial to mesenchymal transition and fibrosis of the intima.

A consequence is the reduced production of the vasodilators prostaglandins and nitric oxide and an increased secretion of the vasoconstrictor endothelin-1 [55]. A prominent interplay between the immune system and the microvasculature characterize the early phase of systemic sclerosis. The initiation and maintenance of the vascular leak is the result of the injury to the vessel wall and endothelium, the loss of the vascular tone control, and the contemporary activation and homing of inflammatory cells. Vascular dysfunction manifested as Raynaud’s phenomenon is the most typical presentation of systemic sclerosis. Morphologic changes of capillaries suggest vascular leak and display functional deficits that result in a progressive hyperpermeable state as described in non-invasive investigational studies. Nailfold videocapillaroscopy (NVC) demonstrates progressive loss of the peripheral vascular network, dilatation of capillaries, deficient vascular repair, and inadequate angiogenesis [56]. Growing evidence demonstrate that systemic sclerosis is primarily a vascular disease where dysfunctions of endothelia are present and prominent in different aspects of cell survival (activation and apoptosis), angiogenesis and vasculogenesis. Furthermore, altered relationships of endothelial cells with various other cells contribute to the vasculopathy. The crucial initiating event that leads to vascular remodeling and capillary breakdown and finish with blood vessel occlusion in scleroderma is the endothelial cell injury [57]. Even if some aspects remain elusive, an initial vascular injury due to autoimmunity and/or environmental factors is recognized as the first contributor to the pathogenesis of the disease. It causes structural and functional abnormalities of the vasculature which eventually induce the constitutive activation of fibroblasts in multiple organs [58]. Microangiopathy and autoantibodies are two aspects characterizing SS and shared with AN. Features of systemic sclerosis are a deficiency of B regulatory cells, the hyperactivation of B cells, the production of profibrotic cytokines such as interleukin-6 and transforming growth factor b and the presence of autoantibodies that can induce fibrosis and vasoconstriction. As described, some of these conditions are reported in AN. AN is associated with a reduction of NK cells and neutrophils and with the impairment of neutrophilic chemotaxis and adherence. Peripheral blood CD8+ cells are reduced with consequent increase of CD4/CD8 T cells ratios while T cell proliferation may result decreased, increased, or unchanged. B cells perform a variety of functions, thus playing a versatile role in humoral immunity with both pro- and anti-inflammatory actions. In particular, J. Freff et al. [59] demonstrated in AN patients a marked reduction in the frequencies of transitional CD24+CD38hi and CD1d+CD5+ B cell subsets. Regulatory B cells perform immunomodulatory activities, maintaining self-tolerance, and consistently with their role, they were found impaired in function and decreased in numbers in autoimmune diseases. In light of this, Freff et al.’s observation suggest a B regulatory cells impairment in AN that support an enhanced possibility to develop autoimmune disorders. The reduced expression of CD5 on transitional B cells [59] and the reduced CD5 plasma level [60] reported in AN patients could impair the maintenance of self-tolerance and survival of B cells.

## 9. Anorexia Nervosa and the Microbiota

Furthermore, a brain and gut healthy communication network cannot disregard microbiota homeostasis. The intestine can interact with the brain through three main mechanisms: the enteric nervous system, the enteroendocrine cells of the gut, and the neurotransmitters produced by the gut microbiota. In particular the enteric nervous system (ENS) is one of the three components of the autonomic nervous system (together with the orthosympathetic and the parasympathetic ones). ENS is an extensive reflex control system for the digestive function that works with the central nervous system and neural pathways that pass through sympathetic ganglia. The ENS interacts with both the gut endocrine and immune systems and has roles in modifying nutrient absorption and maintaining the mucosal barrier [61]. The microbiota supports the ENS formed at birth and participates in its homeostasis throughout adult life. A close correlation between the ENS and the microbiota has been demonstrated and we have to take into account the possibility of a close correlation between the dysfunction of the ENS, the microbiota, and the diseases of the central nervous system [62].

Some authors in the pathophysiological development of eating disorders hypothesized immune reactions triggered in the hunger-satiety regulation center by altered gut permeability promoted by intestinal inflammation due to dysbiosis [63]. Many important players of anorexia nervosa are influenced by gut microbiota such as gut inflammation and permeability, energy food extraction and body weight gain, anxiety, depression, and complex psychological behaviors. The gut microbiota is mainly influenced by nutrition and obviously in AN selective eating and nutritional restriction are relevant influencing factors [64,65,66,67,68,69]. The influence of gut microbiota on host health and physiology and more specifically its role in regulating the dysfunction, as reaction to different injuries, of distant organs and systems has been the subject of numerous studies [70]. A fundamental feature of endothelium function is the mediated nitric-oxide vasodilatation that diet and possibly the gut microbiota could modulate. Prebiotic supplementation can significantly improve vascular function through the nitric oxide pathway [71]. More generally, the study demonstrates the existing relationship between diet, gut microbiota, and the regulation of the host physiological functions [72].

## 10. Conclusions

**Figure 2:**
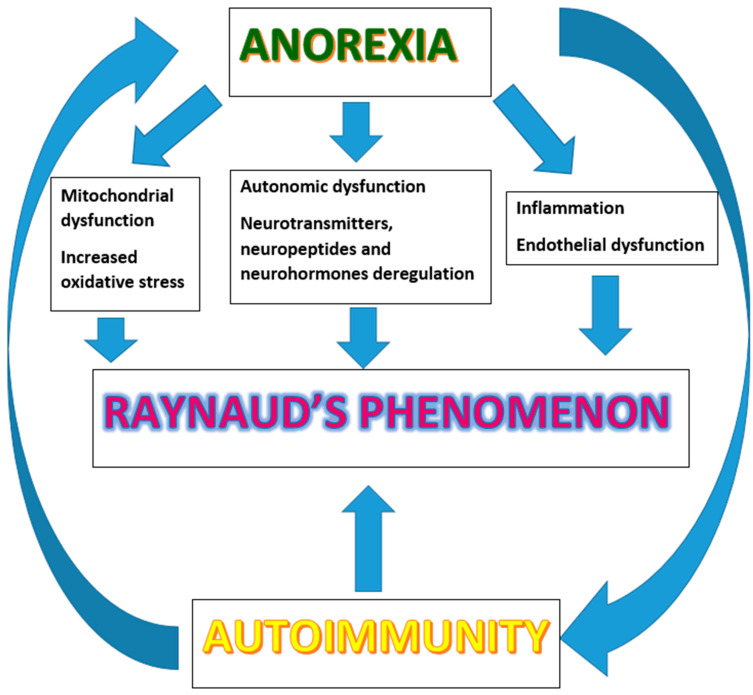
The complex relationship between anorexia nervosa, peripheral vascular abnormalities, and autoimmunity. Psycho-neuro-endocrine-immunology offers a new unifying view of these disorders. Immune, neuroendocrine, and autonomic nervous system dysregulation are described in AN. The presence of Raynaud’s phenomenon as in autoimmune disorders raises several questions. Several mechanisms, as shown, are at the basis of the peripheral vascular abnormalities. There is a bidirectional relationship between autoimmunity and anorexia nervosa: autoimmune diseases and eating disorders give each other a mutual increased risk [42,43].

It is part of our daily clinical practice to follow paths of deductive reasoning, and the classic figure of the syllogism seem to be applicable in the case of AN, peripheral vascular dysfunction, and autoimmunity. We knew that AN is associated to RP and to immune system dysregulation with an increased risk for autoimmune diseases. Several observations, as discussed, let us speculate a common reciprocal pathway on endothelial damage and vascular reactivity between AN and autoimmunity. It is true that we know a little about this intriguing story and it is therefore mandatory to deepen it in a targeted way to test such compelling hypothesis. Clinicians treating patients with eating disorders are recommended to consider the increased risk of autoimmune diseases. Mood disturbances, anxiety, and disordered eating may show neuropsychiatric and somatic symptoms with a possible underlying role of autoimmune processes [52]. Future studies are needed to better explore the immunological mechanisms in individuals with various eating disorders and their family members and the risk they run to develop autoimmune diseases. In this view, we suggest to consider in the diagnostic route, at least in the subset of patients with peripheral microvascular symptoms, a nailfoldvideocapillaroscopy [73,74,75,76,77] as an easy not invasive tool for the early assessing of possible cardiovascular involvement. We have so far described the complex network that links AN, RP, and autoimmunity. Psychoneuroendocrineimmunology offers a new unifying view of these disorders (Figure 2) integrating knowledge derived from the biological and psychological sciences with molecular biology, as well as genetic and epigenetic research. A circular relationship exists between mental disorders and lifestyle behavior via the release of proinflammatory cytokines and changes in the metabolic profile capable of altering the physiological functions of target organs and biological systems [18,78]. In conclusion, we hope that our reflections may offer an additional contribution for further discussion on the origin, pathological mechanism, and possible evolution of peripheral vascular alterations in eating disorders and to their better diagnosis, prevention, and treatment.
